# Prognostic Role of the C-Reactive Protein/Albumin Ratio in Patients With Gynecological Cancers: A Meta-Analysis

**DOI:** 10.3389/fonc.2021.737155

**Published:** 2021-10-28

**Authors:** Yingji Fang, Tingting Zheng, Chengling Zhang

**Affiliations:** Department of Gynecology, Jinan Maternal and Child Care Hospital, Jinan, China

**Keywords:** gynecological cancers, meta-analysis, prognostic, CRP/Alb ratio, risk factors

## Abstract

**Background:**

Many studies have investigated the prognostic role of the C-reactive protein/albumin ratio (CRP/Alb ratio) in patients with gynecological cancers; however, there is lack of consensus owing to conflicting results across studies. We performed a meta-analysis to determine the prognostic role of the CRP/Alb ratio in gynecological cancers.

**Methods:**

We searched the PubMed, Embase, the Web of Science, Cochrane Library, China National Knowledge Infrastructure, and Wanfang electronic databases since inception to April 2021. Combined hazard ratios (HRs) and 95% confidence intervals (CIs) were used to estimate the prognostic effect of the CRP/Alb ratio in gynecological cancers. Pooled odds ratios (ORs) and 95% CIs were used to investigate the association between the CRP/Alb ratio and clinicopathological features.

**Results:**

The meta-analysis included seven studies with 1,847 patients. The pooled results showed that a high pretreatment CRP/Alb ratio was associated with poor overall survival (HR, 1.84; 95% CI, 1.41–2.40; p < 0.001) and progression-/disease-free survival (HR, 2.58; 95% CI, 1.42–4.68; p = 0.002). Additionally, a high CRP/Alb ratio was significantly associated with stages III–IV disease (the International Federation of Gynecology and Obstetrics classification) (OR, 2.98; 95% CI, 1.45–6.14; p = 0.003). However, we observed a non-significant correlation between the CRP/Alb ratio and lymph node metastasis, tumor size, and histopathological grade.

**Conclusions:**

The CRP/Alb ratio is a convenient and accurate predictor of survival outcomes in gynecological cancers. A high CRP/Alb ratio also predicts tumor progression.

## Introduction

Gynecological cancers (GCs) represent the second most common cancer in women worldwide (1). Cervical cancer (CC), ovarian cancer (OC), and endometrial cancer (EC) are the predominant types of GC, which constitute a major public health concern globally (2). The 5-year survival rates in patients with GC are poor despite the availability of well-established surgical and chemoradiotherapeutic approaches (3). For example, the 5-year survival rate in patients with stages III–IV EC was only 17% (4). Therefore, it is important to identify effective novel biomarkers that predict survival outcomes and also establish individualized therapeutic regimens.

Accumulating evidence has shown an association between tumor-induced inflammatory responses and tumor development and progression (5). Reportedly, various inflammatory and immune response biomarkers serve as prognostic factors for solid tumors (6). For example, in a recent study, Marchetti et al. (7) observed that the neutrophil–lymphocyte ratio (NLR) was an independent prognostic marker for progression-free survival (PFS) in patients with high-grade advanced serous ovarian cancer. Another study (8), which recruited 1,266 patients with CC, showed that the platelet–lymphocyte ratio (PLR), NLR, derived NLR, and the PLR + NLR in combination showed equivalent efficacy as prognostic factors for overall survival (OS) in patients with locally advanced CC and are therefore considered promising prognostic biomarkers. The aforementioned studies (7, 8) highlight the role of systemic inflammatory biomarkers in the prognosis of GC. The C-reactive protein (CRP)/albumin ratio (CRP/Alb) is an index based on measurement of serum CRP and Alb levels (9) and is calculated as the serum CRP level divided by the serum Alb level. This ratio was initially proposed to predict outcomes in patients with acute medical admissions (9). In recent years, studies have investigated the utility of the CRP/Alb ratio as a prognostic factor in various cancers (10, 11). Previous studies have reported the independent prognostic value of the CRP/Alb ratio in colorectal cancer (CRC) (12), oral squamous cell carcinoma (13), gastric cancer (14), non-small-cell lung cancer (15), and gallbladder cancer (16). Many studies have also investigated the prognostic utility of the CRP/Alb ratio in patients with GC; however, the results remain inconclusive (17–23). We performed a meta-analysis to systemically investigate the prognostic and clinical significance of the CRP/Alb ratio in GC.

## Materials and Methods

### Study Guidelines and Ethics

This meta-analysis was performed in accordance with the Preferred Reporting Items for Systematic Reviews and Meta-Analyses statement (24). All analyses were based on previously published studies; therefore, ethical approval and informed consent were waived for this study.

### Literature Search

We performed a comprehensive search of the PubMed, Embase, the Web of Science, Cochrane Library, China National Knowledge Infrastructure, and Wanfang electronic databases from inception to April 2021. Based on relevant studies (25, 26), we used the following search terms: gynecological cancer OR gynecological carcinoma OR gynecological neoplasm OR endometrial neoplasm OR endometrial carcinoma OR endometrial cancer OR endometrium cancer OR endometrium carcinoma OR cervical cancer OR cervical carcinoma OR ovarian carcinoma OR ovarian cancer OR uterine neoplasm and C-reactive protein OR albumin OT CAR OR CRP/Alb OR C-reactive protein to albumin ratio, C-reactive protein/albumin ratio and prognosis OR prognostic OR survival OR outcome OR mortality. Both free text and medical subject heading terms were used for the literature search. We selected articles in English and Chinese. References from the identified publications were also retrieved for potential inclusion.

### Inclusion and Exclusion Criteria

Following were the inclusion criteria: (1) histopathologically diagnosed GC; (2) studies that investigated the prognostic role of CRP/Alb ratio for survival outcomes including but not limited to OS, cancer-specific survival (CSS), disease-free survival (DFS), PFS, and recurrence-free survival; (3) availability of hazard ratios (HRs) and 95% confidence intervals (CIs) or sufficient data to calculate these values; and (4) availability of cutoff values of the CRP/Alb ratio. Following were the exclusion criteria: (1) reviews, meeting abstracts, case reports, and letters; (2) unavailability of data to calculate HRs and corresponding 95% CIs; (3) studies in languages other than English or Chinese; and (4) overlapping or duplication of studies.

### Data Extraction and Quality Assessment

Two investigators (YF and TZ) independently screened all retrieved articles and extracted information using a predetermined form. All disagreements were resolved through discussion with a third investigator (CZ). The following data were recorded: first author’s name, year of publication, country, age, cancer type, the International Federation of Gynecology and Obstetrics (FIGO) stage, treatment, study period, cutoff value of the CRP/Alb ratio, method used to determine the cutoff value, duration of follow-up or the last date of follow-up, survival endpoints, and HRs and 95% CIs for OS, PFS, and DFS. HRs and 95% CIs were extracted from multivariable analyses depending on availability; HRs and 95% CIs were extracted from univariate analyses in the remaining cases. Two independent reviewers assessed the methodological quality of the included studies based on the Newcastle–Ottawa Quality Assessment Scale (NOS) (http://www.ohri.ca/programs/clinical_epidemiology/oxford.asp). The NOS consists of the following three dimensions: selection (four stars), comparability (two stars), and outcome assessment (three points). NOS scores range from 0 to 9, and scores ≥6 indicate high-quality studies.

### Statistical Analysis

Combined HRs and 95% CIs were used to estimate the prognostic effect of the CRP/Alb ratio in GC. HR >1 without a 95% CI overlapping 1 indicates that a high CRP/Alb ratio predicts poor prognosis. Heterogeneity among studies was assessed using the Cochrane Q test and the I^2^ statistic. p < 0.10 and/or I^2^ > 50% was interpreted as indicative of significant heterogeneity, and a random-effects model was used in such cases; a fixed-effects model was used in the remaining cases. Subgroup analysis stratified by various factors was performed to determine the source of heterogeneity. Pooled odds ratios (ORs) and 95% CIs were used to determine the association between the CRP/Alb ratio and clinicopathological features. Publication bias was evaluated using Begg’s funnel plots. All statistical analyses were performed using the Stata software, version 12.0 (Stata Corporation, College Station, TX, USA). A p < 0.05 was considered statistically significant.

## Results

### Literature Search

**Figure 1:**
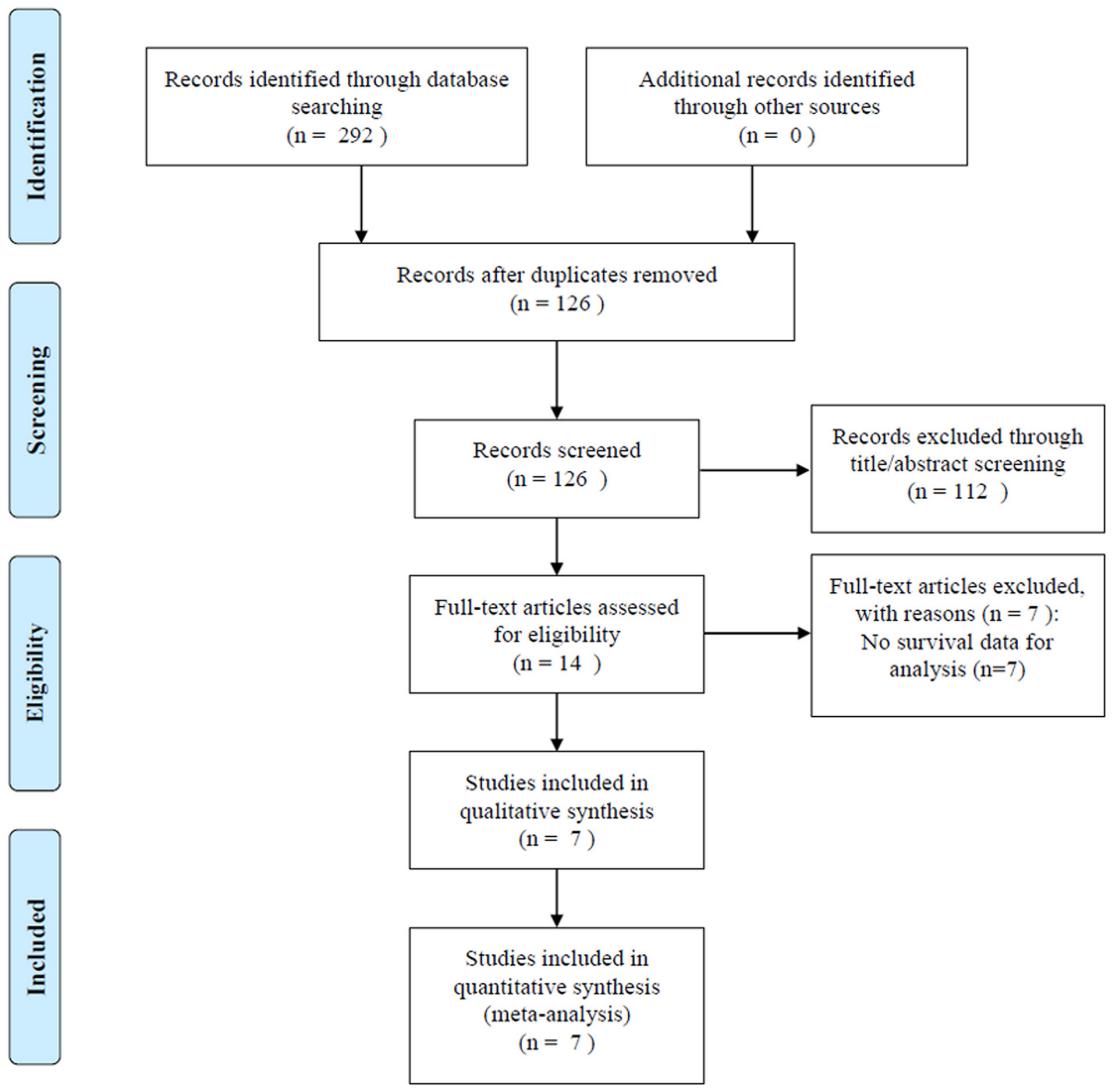
Flowchart describing the literature search and study selection.

The initial literature search identified 292 studies from the aforementioned databases; 126 studies remained after exclusion of duplicate studies (**Figure 1**). After screening titles and abstracts, 112 studies were eliminated, and we reviewed the full text in 14 articles, of which 7 studies with insufficient data were excluded. Finally, seven studies with 1,847 patients (17–23) were included in this meta-analysis.

### Characteristics of Included Studies

**Table 1** summarizes the characteristics of studies included in this meta-analysis. The studies were published between 2017 and 2021. Five studies were performed in China (17, 19, 20, 22, 23) and two in Japan (18, 21). Six studies were published in English (17–19, 21–23) and one in Chinese (20). Four studies enrolled patients with CC (17, 20–22) and three recruited patients with OC (18, 19, 23). The sample size ranged from 200 to 407 (median, 235). The cutoff values of the CRP/Alb ratio ranged from 0.022 to 0.68 (median, 0.15). Therefore, we used 0.15 as the cutoff value of the CRP/Alb ratio for subgroup analysis. Five studies used receiver operating characteristic curve analysis to determine the cutoff value (17–20, 22), one study used the median value (21), and one study used cutoff finder software (23). Six studies (17, 19–23) reported the prognostic value of the CRP/Alb ratio for OS, two studies (22, 23) reported its prognostic value for PFS, and one study (18) for DFS. Five studies (17–19, 21, 23) enrolled patients with FIGO stages I–IV, and two studies (20, 22) included patients with FIGO stages I–II. NOS scores of the included studies ranged from 6 to 9 (median, 7), which indicates that all included studies were of high quality. **Table 2** shows NOS score details.

**Table 1:** Summary of clinical studies included in meta-analysis.

Study	Year	Country	Sample size	Age	Cancer type	FIGO stage	Treatment	Study period	Cutoff value	Follow-up (months) or the date of last follow-up	Determination of cutoff value	Survival endpoints	CRP/Alb value (high/low)	NOS score
He et al. (17)	2018	China	229	44 (28–79)	Cervical cancer	I–IV	Surgery + chemotherapy	2007–2009	0.022	To Jan, 2016	ROC analysis	OS	138/91	7
Komura et al. (18)	2020	Japan	308	NA	Ovarian cancer	I–IV	Surgery + chemotherapy	2007–2016	0.048	NA	ROC analysis	DFS	156/152	6
Liu et al. (19)	2017	China	200	53(18–83)	Ovarian cancer	I–IV	Surgery + chemotherapy	2006–2012	0.68	To Dec, 2014	ROC analysis	OS	69/131	9
Su et al. (20)	2020	China	407	NA	Cervical cancer	I–II	Surgery	2009–2013	0.15	To Apr, 2018	ROC analysis	OS	61/346	7
Taguchi et al. (21)	2021	Japan	231	67	Cervical cancer	I–IV	Radiotherapy	2004-2015	0.18	16.4	Median value	OS	116/115	8
Zhang et al. (22)	2018	China	235	46(29–78)	Cervical cancer	I–II	Surgery	2005–2009	0.15	77(32–96)	ROC analysis	OS, PFS	113/122	7
Zhang et al. (23)	2017	China	237	NA	Ovarian cancer	I–IV	Surgery + chemotherapy	2007–2015	0.5	To Dec, 2016	Cutout finder	OS, PFS	95/142	7

FIGO, International Federation of Gynecology and Obstetrics; NA, not available; OS, overall survival; PFS, progression-free survival; DFS, disease-free survival; ROC, receiver operating characteristics; NOS, Newcastle-Ottawa Scale; CRP/Alb, C-reactive protein/albumin ratio.

**Table 2:** The details of NOS scale for studies in the meta-analysis.

First author	Year		Selection			Comparability		Outcome		Total score
		Representativeness of the exposed cohort	Selection of the non-exposed cohort	Ascertainment of exposure	Demonstration that outcome of interest was not present at start of study	Comparability of cohorts on the basis of the design or analysis	Assessment of outcome	Was follow-up long enough for outcomes to occur	Adequacy of follow up of cohorts	
He et al. (17)	2018	★	★	★	★	★	★	–	★	7
Komura et al. (18)	2020	★	★	–	★	★	★	★	–	6
Liu et al. (19)	2017	★	★	★	★	★★	★	★	★	9
Su et al. (20)	2020	★	★	★	★	★	★	–	★	7
Taguchi et al. (21)	2021	★	★	★	★	★★	★	–	★	8
Zhang et al. (22)	2018	★	★	★	★	★	★	★	–	7
Zhang et al. (23)	2017	★	★	–	★	★★	★	–	★	7

NOS, Newcastle–Ottawa Scale A star is one point.

### Prognostic Role of the C-Reactive Protein/Albumin Ratio in Overall Survival and Progression- and Disease-Free Survival

**Figure 2:**
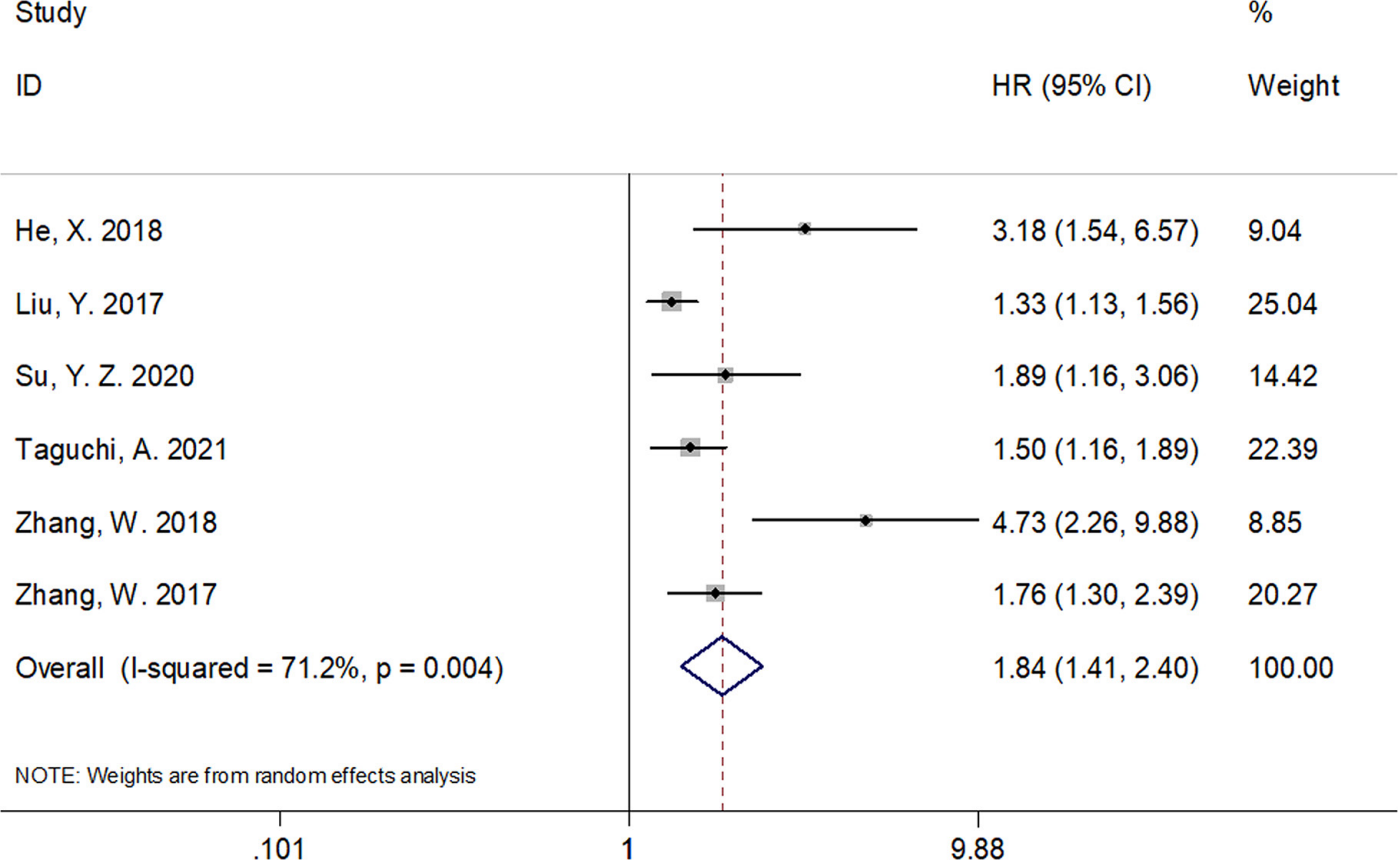
Forest plots of studies evaluating the hazard ratio for overall survival (OS) of patients with gynecological cancers of a high CRP/Alb ratio.

**Figure 3:**
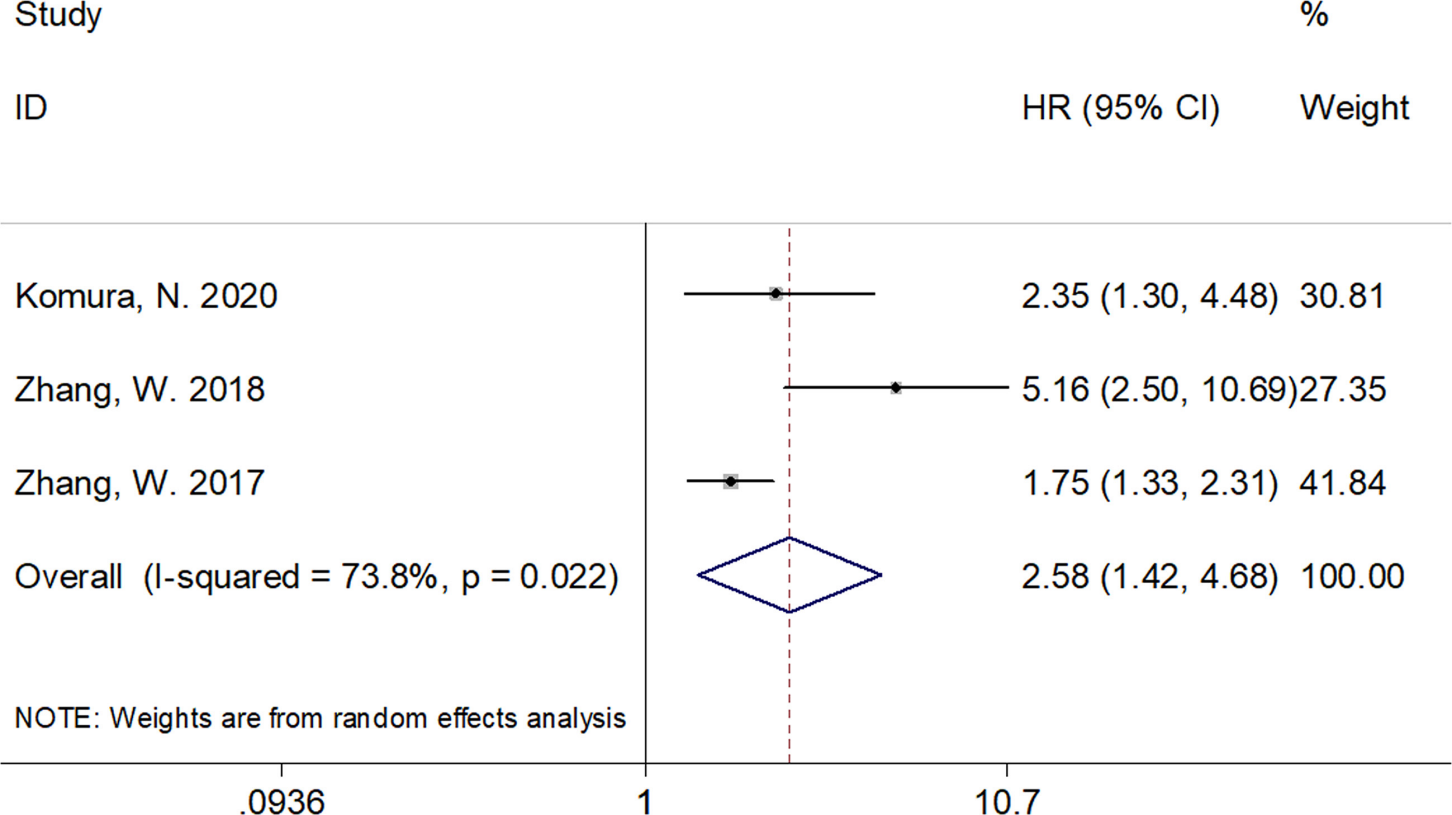
Forest plots of studies evaluating the hazard ratio for progression-free survival (PFS)/disease-free survival (DFS) of patients with gynecological cancers of a high CRP/Alb ratio.

A total of six studies that included 1,539 patients (17, 19–23) investigated the association between the CRP/Alb ratio and OS in GC. Pooled data showed HR of 1.84, 95% CI of 1.41–2.40, and p < 0.001 (**Figure 2**; **Table 3**). Owing to significant heterogeneity (I^2^ = 71.2%, p = 0.004), we used a random-effects model. Subgroup analyses based on various factors showed that a high CRP/Alb ratio remained a prognostic tool to determine poor survival in subgroups across different countries, cancer types, FIGO stage, cutoff values, cutoff value determination methods, and treatment modalities (**Table 3**). Three studies that included 780 patients (18, 22, 23) reported the role of the CRP/Alb ratio for prognosis of PFS/DFS (HR, 2.58; 95% CI, 1.42–4.68; p = 0.002) based on a random-effects model (**Figure 3**; **Table 3**). Similar to the findings associated with OS, subgroup analysis revealed that an elevated CRP/Alb ratio was an indicator of poor PFS/DFS in various subgroups of patients with GC.

**Table 3:** Subgroup analysis of the prognostic value of CRP/Alb for OS and PFS/DFS in patients with gynecological cancers.

Subgroups	No. of studies	No. of patients	Effects model	HR (95% CI)	p	Heterogeneity	
						*I* ^2^ (%)	Ph
OS							
Total	6	1539	Random	1.84 (1.41–2.40)	<0.001	71.2	0.004
Country							
China	5	1308	Random	2.05 (1.42–2.97)	<0.001	76.9	0.002
Japan	1	231	–	1.50 (1.18–1.91)	0.001	–	–
Cancer type							
Cervical cancer	4	1102	Random	2.33 (1.43–3.79)	0.001	73.1	0.011
Ovarian cancer	2	437	Random	1.49 (1.13–1.94)	0.004	61.0	0.109
Tumor stage							
I–II	2	642	Random	2.86 (1.17–7.01)	0.022	76.1	0.041
I–IV	4	897	Random	1.58 (1.27–1.98)	<0.001	58.4	0.066
Cut–off value							
≤0.15	3	871	Random	2.88 (1.65–5.02)	<0.001	55.7	0.105
>0.15	3	668	Fixed	1.44 (1.27–1.63)	<0.001	26.7	0.256
Cutoff value determination							
ROC analysis	4	1071	Random	2.28 (1.31–3.99)	0.004	81.7	0.001
Median value/cutoff finder	2	468	Fixed	1.60 (1.32–1.93)	<0.001	0	0.417
Treatment							
Surgery + chemotherapy	3	666	Random	1.46 (1.27–1.68)	<0.001	72.1	0.028
Surgery	2	642	Random	2.49 (1.66–3.73)	<0.001	76.1	0.041
Radiotherapy	1	231	–	1.50 (1.18–1.91)	0.001	–	–
PFS/DFS							
Total	3	780	Random	2.58 (1.42–4.68)	0.002	73.8	0.022
Country							
China	2	472	Random	2.85 (0.99–8.17)	0.052	86.5	0.006
Japan	1	308	–	2.35 (1.27–4.36)	0.007	–	–
Cancer type							
Cervical cancer	1	235	–	5.16 (2.50–10.69)	<0.001	–	–
Ovarian cancer	2	545	Fixed	1.84 (1.43–2.37)	<0.001	0	0.395
Tumor stage							
I–II	1	235	–	5.16 (2.50–10.69)	<0.001	–	–
I–IV	2	545	Fixed	1.84 (1.43–2.37)	<0.001	0	0.395
Cutoff value							
≤0.15	2	543	Random	3.40 (1.57–7.34)	0.002	61.7	0.106
>0.15	1	237	–	1.75 (1.33–2.31)	<0.001	–	–
Cutoff value determination							
ROC analysis	2	543	Random	3.40 (1.57–7.34)	0.002	61.7	0.106
Median value/cutoff finder	1	237	–	1.75 (1.33–2.31)	<0.001	–	–
Treatment							
Surgery + chemotherapy	2	545	Fixed	1.84 (1.43–2.37)	<0.001	0	0.395
Surgery	1	235	–	5.16 (2.50–10.69)	<0.001	–	–

OS, overall survival; PFS, progression-free survival; DFS, disease-free survival; ROC, receiver operating characteristics.

### Correlation Between the C-Reactive Protein/Albumin Ratio and Clinical Features

**Figure 4:**
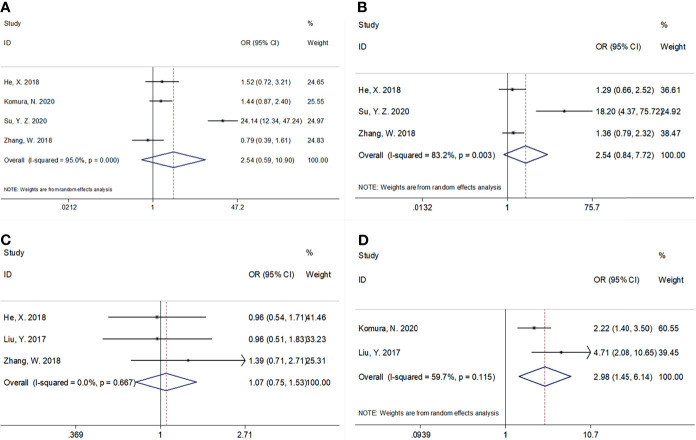
The association between CRP/Alb ratio and clinicopathological features in patients with gynecological cancers. **(A)** Lymph node metastasis (yes vs. no), **(B)** tumor size (≥4 vs. <4 cm), **(C)** histological grade (G3 vs. G1/G2), and **(D)** FIGO stage (III–IV vs. I–II).

Using data from five studies, we investigated the association between the CRP/Alb ratio and clinicopathological characteristics (17–20, 22). A high CRP/Alb ratio was significantly associated with FIGO stages III–IV (OR, 2.98; 95% CI, 1.45–6.14; p = 0.003) (**Figure 4**; **Table 4**). However, we observed a non-significant correlation between the CRP/Alb ratio and lymph node metastasis (yes vs. no) (OR, 2.54; 95% CI, 0.59–10.90; p = 0.209), tumor size (≥4 vs. <4 cm) (OR, 2.54; 95% CI, 0.84–7.72; p = 0.100), and histopathological grade (G3 vs. G1/G2) (OR, 1.07; 95% CI, 0.75–1.53; p = 0.176) (**Figure 4**; **Table 4**).

**Table 4:** The correlation between CRP/Alb ratio and clinicopathological features in patients with gynecological cancers.

Variables	No. of studies	No. of patients	Effects model	HR (95%CI)	p	Heterogeneity	
						*I* ^2^ (%)	Ph
Lymph node metastasis (yes vs. no)	4	1,179	Random	2.54 (0.59–10.90)	0.209	95.0	<0.001
Tumor size (≥4 vs. <4 cm)	3	871	Random	2.54 (0.84–7.72)	0.100	83.2	0.003
Histological grade (G3 vs. G1/G2)	3	664	Fixed	1.07 (0.75–1.53)	0.716	0	0.667
FIGO stage (III–IV vs. I–II)	2	508	Random	2.98 (1.45–6.14)	0.003	59.7	0.115

FIGO, International Federation of Gynecology and Obstetrics.

### Publication Bias

**Figure 5:**
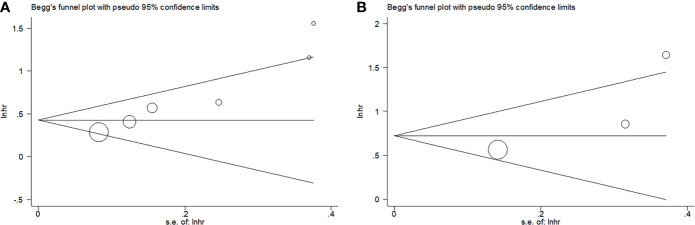
Publication bias tested by funnel plots in this meta-analysis. **(A)** OS; **(B)** PFS/DFS.

Begg’s funnel plots were used to assess the potential publication bias for OS and PFS/DFS. We observed no significant publication bias for OS (p = 0.135) or PFS/DFS (p = 0.296) in this meta-analysis (**Figure 5**). Therefore, the results of our meta-analysis are reliable.

## Discussion

The CRP/Alb ratio has been investigated as a prognostic factor for patients with GC; however, there is lack of consensus owing to inconsistent results across studies. In the present meta-analysis, we used pooled data from seven studies that included 1,847 patients; our results showed that a high CRP/Alb ratio was an independent prognostic factor for poor OS, PFS, and DFS. Results of subgroup analyses performed after stratification based on country, cancer type, FIGO stage, cutoff values, cutoff value determination methods, and treatment modalities were in agreement with the results of the overall analysis. An elevated CRP/Alb ratio was also associated with advanced FIGO stages in GC. These results indicate that the CRP/Alb ratio may serve as an efficient and cost-effective prognostic biomarker in clinical settings for patients with GC. To our knowledge, this is the first meta-analysis that discusses the prognostic role of the CRP/Alb ratio in GC.

Inflammatory responses are known to promote tumorigenesis through their effects on the tumor microenvironment in GC (27). Tumor growth, invasion, necrosis, and hypoxia initiate immune responses in the tumor microenvironment, which consequently triggers the production of a variety of inflammatory cytokines (28). CRP is an acute-phase protein that is mediated by several proinflammatory cytokines, including interleukin-1 (IL-1), IL-6, and tumor necrosis factor-α (29). IL-6 can lead to inflammation and angiogenesis, which contribute to tumor progression (30). Serum Alb levels reflect patients’ nutritional status; a low serum Alb level indicates a state of malnutrition (31). Hypoalbuminemia is implicated in the nutritional decline observed in patients with cancer (17). Therefore, the CRP/Alb ratio (as a combination of CRP and Alb) provides a biological rationale to be considered a promising prognostic tool in GC.

Many studies have investigated the prognostic value of the CRP/Alb ratio in a variety of cancer types (26, 29, 32–35). Yu et al. showed that an elevated pretreatment CRP/Alb ratio was a prognostic marker of poor OS and CSS in patients with gastric cancer (26). A high CRP/Alb ratio was shown to be associated with clinicopathological features that reflect tumor progression in patients with gastric cancer (26). A meta-analysis of 15 studies that included 6,329 patients reported that a high CRP/Alb ratio was associated with various survival outcomes in patients with CRC (29). Another recent meta-analysis suggested that an elevated pretreatment CRP/Alb ratio could independently predict poor OS in patients with pancreatic cancer (33). Zhou et al. observed that a high pretreatment CRP/Alb ratio predicted poor survival in patients with renal cell carcinoma (36). In our meta-analysis, the CRP/Alb ratio was correlated with poor OS, PFS, and DFS and also predicted advanced FIGO stages. Therefore, the CRP/Alb ratio also serves as a promising tool for risk stratification of patients.

Following are the limitations of this meta-analysis: (a) we investigated studies that described only a few types of GC; only CC and OC were considered in this study. Other GCs, such as EC, were not included in our meta-analysis. Although the search items included all GCs; however, no eligible study on EC was identified. (b) All eligible studies were retrospectively designed, which may introduce selection bias in the meta-analysis. (c) All patients were from China and Japan; therefore, our findings may not be generalizable and are perhaps more applicable to Asian patients. The prognostic value of the CRP/Alb ratio in non-Asian patients warrants investigation.

## Conclusions

Our meta-analysis showed that the CRP/Alb ratio is a convenient and accurate predictor of survival outcomes in GC. A high CRP/Alb ratio also predicts tumor progression. However, owing to several limitations of this study, large-scale trials that include patients of diverse ethnicities are warranted to validate our findings.

## Data Availability Statement

The original contributions presented in the study are included in the article/supplementary material. Further inquiries can be directed to the corresponding author.

## Author Contributions

YF and TZ came up with the study. All authors collaborated on the design of the project. YF, TZ, and CZ collated, screened, and analyzed the data together and drafted the manuscript. YF and TZ reviewed the content, revised the manuscript, and approved the final manuscript. All authors contributed to the article and approved the submitted version.

## Conflict of Interest

The authors declare that the research was conducted in the absence of any commercial or financial relationships that could be construed as a potential conflict of interest.

## Publisher’s Note

All claims expressed in this article are solely those of the authors and do not necessarily represent those of their affiliated organizations, or those of the publisher, the editors and the reviewers. Any product that may be evaluated in this article, or claim that may be made by its manufacturer, is not guaranteed or endorsed by the publisher.
